# Optimization of hydrogenobyrinic acid biosynthesis in *Escherichia coli* using multi-level metabolic engineering strategies

**DOI:** 10.1186/s12934-020-01377-2

**Published:** 2020-06-01

**Authors:** Pingtao Jiang, Huan Fang, Jing Zhao, Huina Dong, Zhaoxia Jin, Dawei Zhang

**Affiliations:** 1 School of Biological Engineering, Dalian Polytechnic University, Dalian, 116034 China; 2 Tianjin Institute of Industrial Biotechnology, Chinese Academy of Sciences, Tianjin, 300308 China; 3 Key Laboratory of Systems Microbial Biotechnology, Chinese Academy of Sciences, Tianjin, 300308 China; 4 University of Chinese Academy of Sciences, Beijing, 100049 China

**Keywords:** Hydrogenobyrinic acid, Uroporphyrinogen III, *Escherichia coli*, Metabolic engineering, Ribosomal binding site libraries

## Abstract

**Background:**

Hydrogenobyrinic acid is a key intermediate of the de-novo aerobic biosynthesis pathway of vitamin B_12_. The introduction of a heterologous de novo vitamin B_12_ biosynthesis pathway in *Escherichia coli* offers an alternative approach for its production. Although *E. coli* avoids major limitations that currently faced by industrial producers of vitamin B_12_, such as long growth cycles, the insufficient supply of hydrogenobyrinic acid restricts industrial vitamin B_12_ production.

**Results:**

By designing combinatorial ribosomal binding site libraries of the *hemABCD* genes in vivo, we found that their optimal relative translational initiation rates are 10:1:1:5. The transcriptional coordination of the uroporphyrinogen III biosynthetic module was realized by promoter engineering of the *hemABCD* operon. Knockdown of competitive heme and siroheme biosynthesis pathways by RBS engineering enhanced the hydrogenobyrinic acid titer to 20.54 and 15.85 mg L^−1^, respectively. Combined fine-tuning of the heme and siroheme biosynthetic pathways enhanced the hydrogenobyrinic acid titer to 22.57 mg L^−1^, representing a remarkable increase of 1356.13% compared with the original strain FH215-HBA.

**Conclusions:**

Through multi-level metabolic engineering strategies, we achieved the metabolic balance of the uroporphyrinogen III biosynthesis pathway, eliminated toxicity due to by-product accumulation, and finally achieved a high HBA titer of 22.57 mg L^−1^ in *E. coli*. This lays the foundation for high-yield production of vitamin B_12_ in *E. coli* and will hopefully accelerate its industrial production.

## Background

**Fig. 1:**
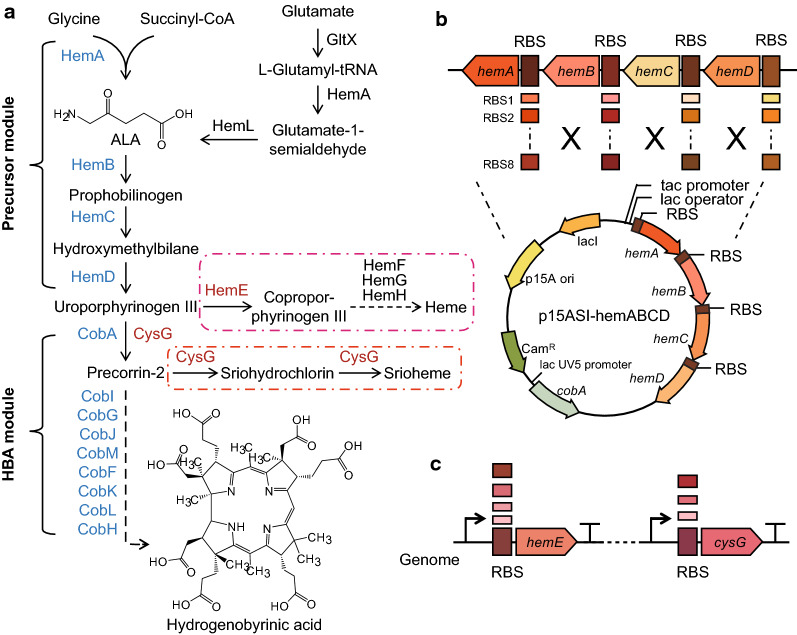
The HBA biosynthesis pathway and related modification strategies. **a** HBA biosynthesis pathway. The introduced foreign genes are marked blue, and the whole pathway was divided into a precursor module and an HBA module. The two branching pathways are shown in the red box. **b** An RBS library and combinatorial constructs were used to screen the optimal relative expression levels for the four heterologous genes *hemABCD* to achieve the purpose of optimizing precursor supply. **c** Weakening the expression of the native *hemE* and *cysG* genes on the chromosome

Hydrogenobyrinic acid (HBA) is the first stable intermediate in the oxygen-dependent (aerobic) biosynthetic pathway of vitamin B_12_ (cobalamin) [1, 2]. It is a modified tetrapyrrole that belongs to the same class of compounds as heme, chlorophyll, siroheme, and coenzyme F430 [3]. Modified tetrapyrroles are synthesized via a branched biosynthetic pathway, with 5-aminolevulinic acid (ALA) as the first common intermediate, and uroporphyrinogen (urogen) III as the first branch-point intermediate in the pathway (Fig. 1a) [2]. The transformation of urogen III into HBA, which was elucidated in 1990s, involves 8 *S*-adenosyl-l-methionine (SAM)-dependent methylations, extrusion of an integral macrocyclic carbon atom (ring contraction), decarboxylation of an acetate side chain, and methyl migration (Fig. 1a) [4]. In 2012, HBA was synthesized in *E. coli* with the aim of isolating intermediates of cobalamin biosynthesis [5]. Recently, breakthroughs have been made in the field of vitamin B_12_ biosynthesis. Fang et al. achieved the de novo biosynthesis of vitamin B_12_ in *E. coli*, offering a promising alternative route for industrial application [6]. There is no doubt that *E. coli* strains with high HBA production play an important role and have great potential for vitamin B_12_ production.

Many different metabolic engineering strategies have been adopted to increase the yield of tetrapyrrole compounds in previous studies. Kiatpapan and Murooka [7], as well as Piao et al. [8] obtained a recombinant strain which accumulated larger amounts of ALA and porphobilinogen (PBG) by overexpressing the *hemA* gene from *Rhodobacter sphaeroides*, which encodes ALA synthase, and the *hemB* gene from *P. freudenreichii*, which encodes PBG synthase. Previous studies have also shown that an increase of intermediates in the branched biosynthesis pathway of vitamin B_12_, such as ALA, PBG, urogen III and precorrin-2, can increase vitamin B_12_ production [9, 10]. The yield of vitamin B_12_ was increased by overexpressing key genes, modifying the properties of the involved enzymes, reducing the flux through competing pathways. and eliminating feedback inhibition in *Bacillus megaterium* [11]. Zhao et al. successfully increased the yield of free heme in *E. coli* by screening overexpression targets in the heme biosynthesis pathway, regulating the metabolic flux in heme biosynthesis, and disrupting the putative heme degradation enzyme encoded by the *yfeX* gene [12].

In our previous study, we introduced an exogenous urogen III biosynthesis pathway into *E. coli* to realize the biosynthesis of vitamin B_12_, and an siRNA strategy was used to knock down the heme branch pathway [6]. Although these strategies were effective to some extent, they have certain limitations. Repurposing a microbe’s delicate self-regulating native metabolism through the introduction of heterologous pathways frequently causes significant imbalances of pathway fluxes, and reestablishing an optimal pathway balance is vital for successful strain engineering [13, 14]. To further improve the target product yield, it is necessary to balance the metabolic flux through fine adjustment of the biosynthetic pathway. However, traditional genetic engineering approaches have shortcomings such as the narrow range for gene knockdown, and maintenance of regulatory genetic elements on a plasmid. Fine-tuning of target genes via ribosome binding site (RBS) engineering is therefore often preferable. A further enhancement of HBA production thus requires precise tuning of the complex metabolic network cantered around the precursor urogen III, as well as the competing heme and siroheme pathways at transcriptional and translational levels.

In this study, we used a variety of strategies to improve the biosynthesis of HBA in *E. coli*, including the construction and optimization of the biosynthetic pathway of the precursor urogen III, as well as the regulation of the related metabolic fluxes. Specifically, we introduced a heterologous urogen III biosynthesis pathway from *Sinorhizobium meliloti* 320, which was optimized by RBS engineering. Then, the precursor module was coordinated the transcriptional level by promoter engineering. Subsequently, the genes involved in the competing heme and siroheme biosynthesis pathways were finely adjusted by RBS engineering to avoid carbon loss. Finally, through the combination of various strategies, we constructed a recombinant *E. coli* strain with an HBA titer of 22.57 mg L^−1^. The successful construction of a strain that accumulates the precursor HBA paves the way for the subsequent industrial production of vitamin B_12_ in *E. coli*.

## Materials and methods

### Plasmids, strains, media and growth conditions

The strains and plasmids used in this study are described in Additional file 1: Table S1. The primers used in this study are listed in Additional file 1: Table S2. *E. coli* 1655 (DE3) [6] was used as the parental strain for de novo production of HBA, and *E. coli* DH5α was used as the host for plasmid construction. Plasmids pET28a and p15ASI [6] were used for plasmid construction and protein expression.

*Escherichia coli* strains were routinely grown in liquid Luria–Bertani (LB) medium or on LB agar plates with appropriate antibiotics at 37 °C. For sirohydrochlorin production, a single colony was transferred into a 96-deep-well plate with 500 μL LB medium per plate and grown overnight at 37 °C and 700 rpm. Then, the overnight culture was used to inoculate a 96-deep-well plate containing 500 μL 2 × YT medium at an inoculation rate of 1%, and cultured at 37 °C and 700 rpm. When the OD_600_ of the cultures reached 0.6–0.8, IPTG was added to a final concentration of 0.6 mM, and the cultures were incubated further at 32 °C for 8 h. HBA production was conducted as described before [6]. Briefly, seed cultures of recombinant *E. coli* strains were grown overnight in 5 mL of LB medium at 37 °C and 220 rpm. Then, the seed cultures were used to inoculate 250 mL shake-flasks containing 50 mL of TYG medium (5 g L^−1^ yeast extract, 10 g L^−1^ tryptone, 5 g L^−1^ KH_2_PO_4_, 2 g L^−1^ glycine, 10 g L^−1^ succinic acid, 20 mg L^−1^ CoCl_2_·6H_2_O), supplemented with 10 g L^−1^ glucose and appropriate antibiotics, at an inoculation rate of 5%, and grown at 37 °C in a rotary shaker at 220 rpm. When the OD_600_ of the cultures reached 0.8, IPTG was added to a final concentration of 1 mM. The cultures were then incubated at 32 °C for another 24 h. All fermentation experiments were conducted in three biological replicates.

### Construction of combinatorial RBS libraries

The RBS were designed using RBS calculator [15]. According to the requirements, we designed RBS sequences for *hemA*, *hemB*, *hemC* and *hemD*, respectively. In each library, translation initiation rates ranging from 1000 to 100,000 were included, and the library size of each gene was set to 8. The details of the RBS sequences are summarized in Additional file 1: Table S3.

**Fig. 2:**
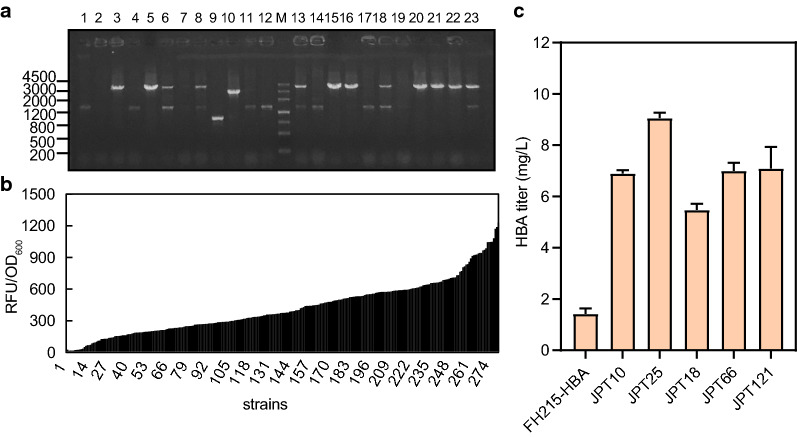
Optimization of the relative expression ratios of ALAS, PBGS, PBGD and UROS using RBS libraries. **a** Colonies were randomly selected and analyzed by PCR to determine the successful assembly of p15ASI-hemABCD. M marker, 23 colonies were selected from an LB plate. **b** The results of 96-deep-well plate fermentation of 288 positive strains. The Y axis represents the fluorescence intensity of the fermentation broth excited at 378 nm, and the strains are arranged according to the fluorescence intensity from low to high. **c** Evaluation of the HBA production capacity of five strains with the highest fluorescence value. Samples of the fermentation broth for HBA analysis were taken at 20 h after induction. Error bars indicate standard deviations from three biological replicates

For the construction of the combinatorial library, degenerate primers with the RBS library sequences attached to the 5′ end were designed and used to amplify the *hemA*, *hemB*, *hemC*, and *hemD* genes. The *Bsa*I restriction sites in the target genes were removed by site-directed mutagenesis to simplify the subsequent gene assembly. Next, the gene *SmcobA* was inserted into p15ASI between the KpnI and XhoI restriction sites, which was confirmed by colony PCR and DNA sequencing, resulting in the plasmid p15ASI-SmcobA, which was used to amplify the vector backbone fragment using the primer pair p5ASISmcobA-golden-F/R. Finally, the five fragments p15ASI-SmcobA, hemA, hemB, hemC and hemD were fused in one step using Golden gate assembly to obtain expression plasmids of the combinatorial RBS library. Then, colony PCR was performed by amplifying the region from *hemA* to *hemD*, with an expected product size of 3.8 kb (Fig. 2a). The RBS sequence of *hemD* on plasmid JPT25 was replaced through site-directed mutagenesis to obtain the plasmids p15ASI-25D2, p15ASI-25D3, p15ASI-25D4 and p15ASI-25D5. The modification of *hemB* was done using the same strategy.

### Construction of the promoter library

A DNA fragment containing the J23119 promoter from the Anderson library, lacUV5 promoters and a bidirectional terminator was synthesized by GENEWIZ (China). This fragment was ligated into an amplified pACYCDuet-1 backbone via Gibson assembly [16], yielding p15AS. The eGFP CDS and RBS were cloned between the PstI and BamHI sites of p15AS, yielding p15AS-eGFP. The primer pair ProLib-F/R was used to build the first promoter library covering promoter mutants with the −35 box and −10 box via site-directed mutagenesis [17]. The primer pair ProLib-F1/R1 was used to build the second promoter library covering promoter mutants with the spacer region via blunt-end cloning.

### RNA preparation and qRT-PCR

Total RNA from cultures of FH215-D2B6, FH215-trc, FH215-F19 and FH215-J23119 was isolated using the RNAprep Pure Cell/Bacteria Kit (TIANGEN, Beijing, China) according to the manufacturer’s instructions. RNA quality and quantity were analyzed using a NanoDrop ND2000 ultramicro spectrophotometer (Thermo Scientific, USA). The cDNA was synthesized from the total RNA using the All-in-One First-Strand cDNA Synthesis SuperMix for qPCR (TransGen, Beijing, China), and qPCR was performed with NovoStart^®^ SYBR qPCR SuperMix Plus (Novoprotein, Beijing, China) according to the manufacturer’s instructions. A negative control without added cDNA was used to check the purity of samples for contaminating genomic DNA. All qPCR assays were performed in triplicate on a 7500 Fast Real-Time PCR System (Applied Biosystems, USA). The cycling parameters were as follows: denaturation step at 95 °C for 1 min followed by 40 cycles of 95 °C for 20 s and 60 °C for 1 min.

### Genome editing in *E. coli*

Genome editing of *E. coli* was based on the CRISPR/Cas9 method reported by Zhao et al. [18]. For example, the up- and downstream homologous arms of *cysG* were amplified, and fused by overlap-extension PCR, while the vector backbone was divided into two fragments with the N20 guide RNA sequence between them. Finally, the three resulting fragments were fused by Golden Gate assembly [19] to create pCas9-DEcysG, which was then introduced into strain FH215 by electroporation, and a positive single colony was transferred to LB medium containing 10 mg L^−1^ arabinose and 100 μg mL^−1^ ampicillin, followed by cultivation at 30 °C for several generations to promote the integration of the construct into the genome. Then, colony PCR and DNA sequencing were used to confirm that *cysG* was deleted successfully. Finally, the *cysG* deleted strain was transferred into LB medium and cultured at 37 °C to cure the pCas9 plasmid, resulting in the final strain FH215DEcysG. The *hemE* weakened strains FH215E100, FH215E500, FH215E1000, FH215E2000, FH215G100, FH215G500, FH215G2000, and FH215E1G2, were constructed using the same method.

### Analytical methods

Cell density was detected by measuring the optical density at 600 nm (OD_600_) using an Ultrospec 3000 spectrophotometer (Amersham Biosciences). Sirohydrochlorin fluorescence was determined using an excitation wavelength of 378 nm and emission wavelength of 600 nm on a Spectra Max M5 instrument (Molecular Devices, USA) [20, 21].

Hematin chloride from Solarbio (China) was used as the analytical standard for heme. The cells from 1 mL of culture broth were separated by centrifugation at 15,700 g and 4 °C for 10 min, and the supernatant was collected to determine the concentration extracellular free heme. The pellet was resuspended in 1 M NaOH and the cells were ultrasonicated on ice using a VCX800 instrument (Sonics, Newtown, USA) equipped with a 3-mm tapered microtip probe set at 30 W for 10 min with 5-s on/5-s off pulse cycles. The insoluble fraction was removed by centrifugation at 15,700*g* and 4 °C for 10 min, and the supernatant was used to determine the concentration of intracellular free heme.

The amount of heme was quantified using high performance liquid chromatography (HPLC; 1260 Infinity II, Agilent, USA) equipped with a diode array detector. A reverse-phase Agilent TC-C18 column (5 μ m, 4.6 mm × 250 mm) was used and the absorbance at 400 nm was monitored. The elution method was modified according to a previous report [12]. For mobile phase, 30% methanol with 0.1% trifluoroacetic acid was used for the first 1 min with a flow rate of 0.8 mL min^−1^. Then, water in the mobile phase was gradually substituted with methanol for the next 10 min, and the buffer composition was maintained for the following 9 min. For the next 3 min, the mobile phase composition gradually changed to the initial ratio, and was maintained for the following 5 min.

### Quantification of hydrogenobyrinic acid

The cells from 5 mL of culture broth were collected by centrifugation at 15,700*g* and 4 °C for 10 min, and suspended in 1.3 mL of sterile water. The samples were placed in a water bath at 100 °C for 30 min, after which the insoluble part was removed by centrifugation at 15,700*g* and 4 °C for 10 min, and the supernatant was used to detect the production of intracellular HBA.

HBA was measured using an Agilent 1260 HPLC system equipped with a diode array detector and an SB-Aq column (4.6 × 150 mm, 5 µm, Agilent). Samples were analyzed at 30 °C and signals were monitored at 329 nm. Samples containing HBA were filtered using a 0.22 µm pore-size inorganic filter, and a 20 µL portion was directly analyzed via HPLC. The mobile phase was water (solvent A) and methanol (solvent B) (both containing 0.1% formic acid) at a flow rate of 1 mL/min. The elution gradient encompassed 0–25% B (0–2 min), 25–34% B (2–4 min), 34–70% B (4–12 min), 70–100% B (12–17 min), 100% B (17–23 min), 0% B (23–25 min), and 0% B (25–32 min) [6].

## Results

### Optimizing the expression ratio of ALAS, PBGS, PBGD and UROS using RBS Libraries

Urogen III is the common precursor of vitamin B_12_, heme and siroheme. Therefore, engineering a strain with high HBA titer for vitamin B_12_ production requires a delicate balance of the metabolic fluxes in the three corresponding pathways. The precursor aminolevulinic acid (ALA) is synthesized by either the C_4_ pathway from glycine and succinyl-CoA catalyzed by 5-aminolevulinic acid synthase (ALAS) or the C_5_ pathway from glutamate through three enzymatic reactions [22]. Urogen III is formed from eight ALA molecules in three consecutive enzymatic steps (Fig. 1a) [23]. Firstly, two ALA molecules are asymmetrically condensed by the enzyme porphobilinogen synthase (PBGS) to form the pyrrole derivative porphobilinogen (PBG). Next, four PBG molecules are linearly linked by the enzyme porphobilinogen deaminase (PBGD) to produce the linear tetrapyrrole pre-uroporphyrinogen (1-hydroxymethylbilane). Finally, urogen III is formed from pre-uroporphyrinogen under the action of uroporphyrinogen III synthase (UROS) [24]. First, the *hemABCD* genes from *S. meliloti* 320, a strain with a high vitamin B_12_ titer [25], were heterologously expressed to supply sufficient urogen III precursor for HBA production. We designed RBS libraries for *hemA*, *hemB*, *hemC*, and *hemD* that covered a broad range of translational initiation rates (TIRs) from around 1000 to 10,000 using RBS Calculator to coordinate the expression of these four genes. The coding sequences were amplified using respective primers with different RBS sequences, and cloned into the p15ASI-SmcobA plasmid expressing the *S*-adenosyl-*l*-methionine dependent uroporphyrinogen III methyltransferase (SUMT) via Golden gate assembly. Consequently, *hemABCD* were expressed as a single operon driven by the tac promoter (Fig. 1b). The enzyme SUMT expressed from the p15ASI-SmcobA plasmid can convert urogen III to precorrin-2, which is then oxidized to sirohydrochlorin. The latter is a fluorescent compound which has an absorption maximum at 378 nm [26], allowing the instable urogen III to be quantified. Various RBS sequences with 8 translational levels were designed for each of the four genes, generating a possible library of 8^4^ mutants. Finally, 288 positive clones were selected for fermentation in 96-deep-well plates.

The relative fluorescence intensity of the selected strains in the fermentation showed a 10-fold range, indicating that the combinatorial RBS libraries yielded a large number of functional constructs with different urogen III production levels (Fig. 2b). Then five plasmids with the highest fluorescence level were selected and used to co-transform the parental strain FH215 together with pET28-HBA, resulting in the recombinant strains JPT10, JPT18, JPT25, JPT66, and JPT121 (Additional file 1: Table S1). These strains were subjected to shake-flask fermentation, and their HBA contents were quantified by HPLC. The HBA titer showed the same trend as the fluorescence intensity of sirohydrochlorin, indicating the direct effect of increased urogen III biosynthesis on HBA production. This result also indicated that it is feasible to evaluate the strength of the precursor pathway via the sirohydrochlorin fluorescence intensity. The HBA titer of the best strain JPT25 reached 9.06 mg L^−1^, representing a 5.39-fold increase over the control strain FH215-HAB, which did not have an exogenous precursor module (Fig. 2c).

### Identifying the optimal expression ratio of ALAS, PBGS, PBGD and UROS in vivo

**Fig. 3:**
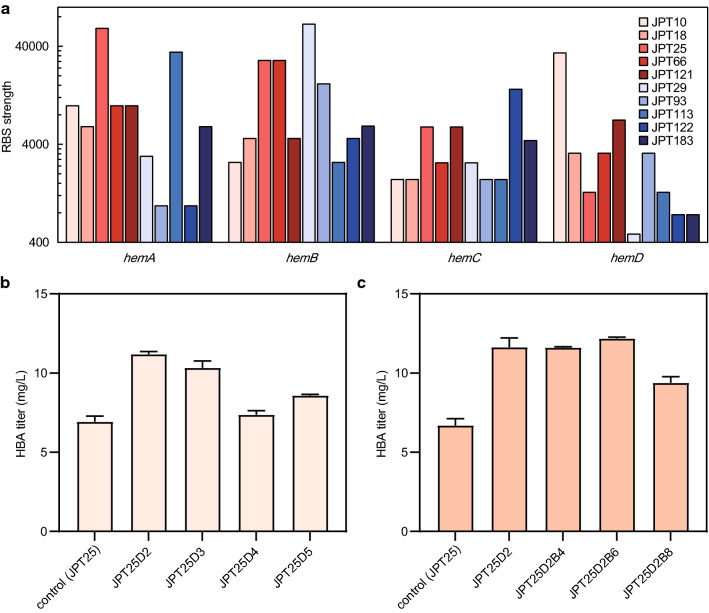
Further optimization of the expression rates of ALAS, PBGS, PBGD and UROS in vivo. **a** Analysis of RBS intensity of five strains with high fluorescence (red series) and five low fluorescence strains (blue series). **b** Change of the HBA titer after optimizing the expression of *hemD* on plasmid p15ASI-25. From JPT25D2 to JPT25D5, the RBS intensity decreased gradually, and all intensities were higher than that of the original RBS. **c** Change of the HBA titer after optimizing the expression of *hemB* on plasmid p15ASI-25D2. From JPT25D2 to JPT25D2B8, the RBS intensity decreased gradually. Error bars indicate standard deviations from three biological replicates

Given that the number of collected strains was much lower than the predicted library capacity, a second round of optimization had to be conducted to obtain a more optimal expression ratio for each gene. Therefore, the RBS of the 5 plasmids with the highest and lowest fluorescence levels were sequenced. The translational intensity of each gene involved in urogen III biosynthesis from the 10 strains was inferred from the original data of the designed RBS library (Additional file 1: Table S4). For easier comprehension, the RBS strength for each gene was given a number from 1 to 8, with a larger number corresponding to a lower RBS strength (Additional file 1: Table S3). Strains with high fluorescence generally showed high translational levels of *hemA* and *hemD*, while most of the strains with low fluorescence intensity showed low translational levels of *hemD* (Fig. 3a). According to these results, we chose the plasmid JPT25, with sufficiently high expression of *hemA*, as the starting point for manual adjustment. First of all, we selected a gradient of enhanced RBS sequences to modify the plasmid, resulting in p15ASI-25D2, p15ASI-25D3, p15ASI-25D4 and p15ASI-25D5. These plasmids were used to co-transform the strain FH215 together with the plasmid pET28-HBA to obtain the HBA-producing strains JPT25D2, JPT25D3, JPT25D4, and JPT25D5 (Additional file 1: Table S1). These strains were evaluated in shake-flask fermentations, and the results showed that the highest HBA yield (11.2 mg L^−1^) was obtained when the translation initiation intensity of *hemD* was 34,133 (Fig. 3b). Obviously, the expression level of *hemD* limits the efficiency of the precursor module, and the production of HBA has been further improved by upregulating its translational level.

The distinct *hemB* translational level of the strains with high fluorescence also attracted our attention, and we weakened the translational level of *hemB* in p15ASI-25D2. The fermentation evaluation of the sequentially *hemB*-weakened strains JPT25D2B4, JPT25D2B6, and JPT25D2B8 showed that *hemB* had a slight influence on the pathway. Only the recombinant strain JPT-25D2B8 showed a certain decline of the HBA titer due to the low translation efficiency of of *hemB*, and only reached 80.77% of the control strain JPT25-D2. When the *hemB* translation efficiency of the recombinant strain JPT-25D2B6 was decreased to 1568.79, the HBA titer was still at the same level as that of the control strain, reaching 12.19 mg L^−1^ (Fig. 3c). It was clear that this strain had lower energy consumption than the control strain while retaining the same production capacity.

At the same time, we constructed the recombinant strains FH215-D2, FH215-D3, FH215-D4, FH215-D5, FH215-D2B4, FH215-D2B6 and FH215-D2B8 only containing the precursor modules. The fluorescence value of sirohydrochlorin in these strains was determined to monitor the production of urogen III indirectly. The results of fluorescence determination showed a similar trend with the HBA titer of each strain (Additional file 1: Fig. S1).

### Optimization of promoters to further increase the yield of hydrogenobyrinic acid

**Fig. 4:**
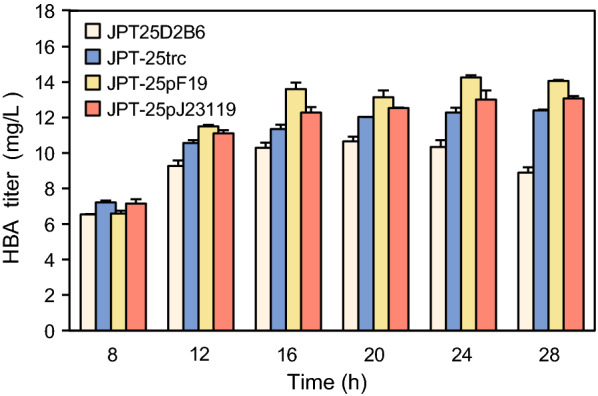
HBA production of the strain with a new promoter in the precursor module. In the control strain JPT25D2B6, *hemABCD* were driven by tac promoter. Recombinant strains JPT25-trc, JPT25-pF19, and JPT25-pJ23119 carried trc, F19 and J23119 promoters, respectively. Error bars indicate standard deviations from three biological replicates

After the optimal ratio of the four enzymes was determined, we investigated whether strengthening the expression of the enzymes at the translational level would further improve HBA production. A variety of constitutive promoters based on the J23119 promoter from the Anderson library were obtained, covering relative GFP strengths ranging from 90.18 to 1467.98. The promoter F19 with the highest strength was 89% stronger than the J23119 promoter. Then, the promoter of the *hemABCD* operon was exchanged with stronger promoters, including inducible promoters trc, as well as the constitutive F19 and J23119 promoters to further increase the expression of the operon. The resulting plasmids p15ASI-25trc, p15ASI-25pF19, and p15ASI-25pJ23119 were introduced into FH215 with the plasmid pET28-HBA, yielding the recombinant strains JPT25-trc, JPT25-pF19, and JPT25-pJ23119 (Additional file 1: Table S1). These strains were subjected to shake-flask fermentation, and their HBA yields were measured from 8 to 28 h. The strain with the *hemABCD* operon driven by the F19 promoter had the highest HBA titer of 14.24 mg L^−1^, which was 1.34-fold higher than that of the control strain JPT-25D2B6 (Fig. 4). The levels of urogen III were measured in the recombinant strains FH215-D2B6, FH215-trc, FH215-F19, and FH215-J23119 containing only precursor modules, and it was found that there was no significant difference in urogen III production among the different strains (Additional file 1: Fig. S2). We speculated that the expression of *cysG* restricted the synthesis of sirohydrochlorin, which led to a certain deviation in the indirect quantification of urogen III levels. As CysG is an enzyme of a competing metabolic branch, it is obviously unwise to overexpress *cysG*. Therefore, in the following experiments, the HBA titer was used as the only evaluation standard for subsequent engineered strains.

At the same time, qRT-PCR was used to investigate the regulation of these promoters and it was found that the expression levels of *hemABCD* genes in these strains were indeed increased. Among them, the transcriptional levels of four *hemABCD* genes driven by the F19 promoter were respectively 18-, 25-, 32- and 8.8-fold higher than those of the original strain with the tac promoter (Additional file 1: Fig. S3).

### Effect of knocking down the heme biosynthesis pathway on HBA production

The metabolic flux towards heme should be reduced because it competes with HBA for common precursors. However, as heme participates in a variety of crucial metabolic processes such as electron transport [27], completely blocking the heme biosynthesis pathway would be lethal. Urogen III is converted to heme through four enzymatic steps. Uroporphyrinogen III decarboxylase (UROD) catalyzes the first step, encompassing the stepwise decarboxylation of the four acetic acid chains of urogen III to yield the corresponding methyl groups of coproporphyrinogen III [24]. We attempted to fine-tune the expression of *hemE*, which encodes UROD, to control heme flux without negatively affecting cell growth. The TIR of the original *hemE* RBS in the genome was predicted by RBS Calculator to be 4785.35. Four RBS were designed for *hemE* using RBS Calculator, to yield lower TIRs of 100, 500, 1000, and 2000 (Additional file 1: Table S3). Then, the native RBS of *hemE* was replaced via CRISPR/Cas9 to generate the *hemE* knockdown strains FH215E100, FH215E500, FH215E1000, and FH215E2000.

**Fig. 5:**
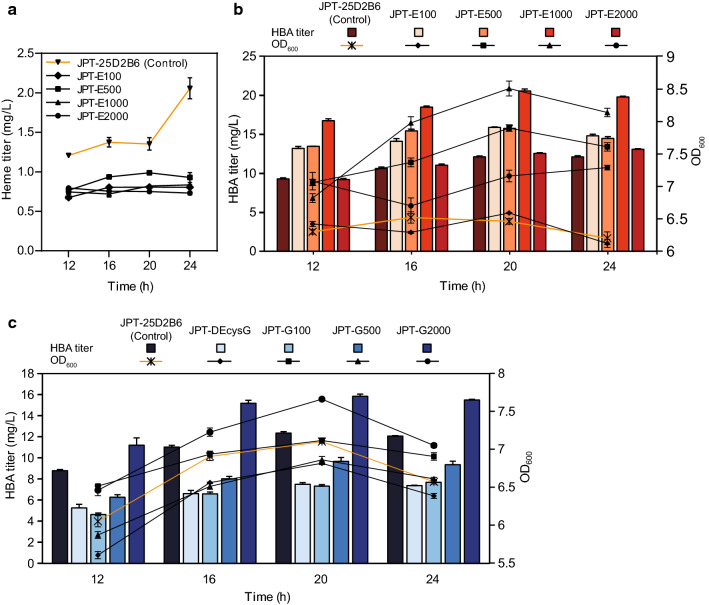
Effect of modified metabolic branch on HBA biosynthesis. **a, b** Effect of weakening the heme pathway on HBA biosynthesis. The intracellular heme concentration (**a**), HBA titer and growth status (**b**) of each strain from 12 to 24 h after induction. **c** Effect of weakening the siroheme pathway on HBA biosynthesis. The HBA titer and growth status of each strain from 12 h to 24 h after induction. The *hemE* knockdown strains: JPT-E100, JPT-E500, JPT-E1000, JPT-E2000. The *cysG* knockdown strains: JPT-G100, JPT-G500, JPT-G2000. The *cysG* knockout strain: JPT-DEcysG. Control: JPT25D2B6. Error bars indicate standard deviations from three biological replicates

The plasmids p15ASI-25D2B6 and pET28-HBA then used to co-transform these *hemE* knockdown strains, yielding the recombinant HBA production strains JPT-E100, JPT-E500, JPT-E1000, and JPT-E2000 (Additional file 1: Table S1). The heme contents of all four strains decreased significantly compared with the control strain, and remained stable after 24 h of fermentation (Fig. 5a). At the same time, the growth status of all parental strains with *hemE* knockdown was better than that of the control strain (Fig. 5b), which can be explained by the elimination of the toxic effect of heme and its intermediates at high concentrations [28, 29]. Among these strains, the HBA titer of JPT-E1000, with an RBS intensity of 1000, was increased by 69.89% compared with the original strain, reaching 20.54 mg L^−1^. The HBA titers of JPT-E100 and JPT-E500 also increased to a certain extent, while the titer of strain JPT-E2000 was basically the same as that of the original strain, which may be caused by insufficient branch weakening (Fig. 5b). These results demonstrated that fine-tuning the expression of genes at the translational level is a powerful approach for achieving both a high product titer and optimal cell growth.

### Effect of knocking down the siroheme biosynthesis pathway on HBA production

Siroheme synthesis also competes for precursors with HBA. It is synthesized from urogen III in three consecutive enzymatic steps via the intermediates precorrin-2 and sirohydrochlorin [30]. In *E. coli*, the SAM-dependent methylation of urogen III, the NAD^+^-dependent dehydrogenation of precorrin-2, and iron insertion into sirohydrochlorin are catalyzed by the multifunctional enzyme CysG [31]. Therefore, knocking out or downregulating *cysG* can block or weaken siroheme synthesis. We obtained a series of mutants by knocking out *cysG*, or by tuning its expression by modifying the RBS sequence (Table S3). The plasmids p15ASI-25D2B6 and pET28-HBA were used to co-transform these mutants, resulting in the strains JPT-DEcysG, JPT-G100, JPT-G500, and JPT-G2000, respectively. Although the *cysG* knockout strain was able to survive in complex medium, the deletion of *cysG* compromised cell viability and decreased the HBA titer markedly. The *cysG* knockdown strains JPT-G100 and JPT-G500 showed similar HBA titers to that of the *CysG* deletion strain, and were down-regulated to a certain extent compared to the control strain. The HBA titer of the strain JPT-G2000 with RBS strength of 2000 increased by 28.34% compared with the control strain JPT-25D2B6, reaching 15.85 mg L^−1^(Fig. 5c).

### Construction of a recombinant *E. coli* strain for HBA production

**Fig. 6:**
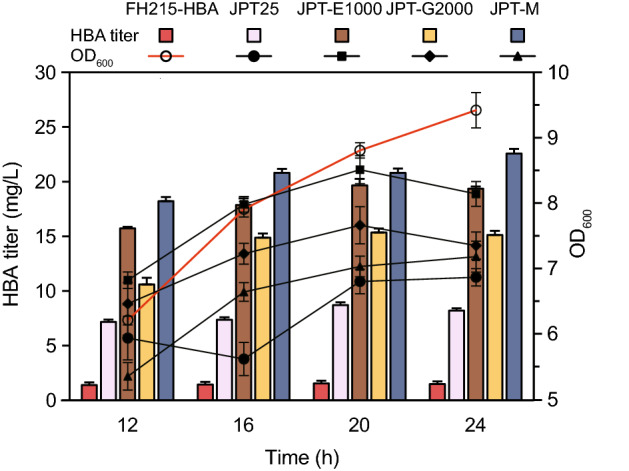
Fermentation of the optimized strain JPT-M, which was constructed by combining multiple strategies. The strains for fermentation in the same batch also include: the control strain FH215-HBA, containing exogenous precursor module strain JPT25, *hemE* knockdown strain JPT-E1000, *cysG* knockdown strain JPT-G2000. Error bars indicate standard deviations from three biological replicates

In the previous experiments, we demonstrated that several different genetic modifications, including fine-tuning of the heme and siroheme biosynthesis pathways, individually led to a significant increase of HBA production. To test whether combined fine-tuning of the heme and siroheme biosynthetic pathways could further facilitate HBA production, we constructed the strain FH215-E1G2 by introducing precise knockdowns of both *hemE* and *cysG* in the chassis strain FH215. Co-transformation of FH215-E1G2 with p15ASI-25pF19, which contains an engineered urogen III biosynthesis pathway with optimal relative expression of individual enzymes, together with pET28-HBA, resulted in the strain JPT-M. The HBA titer of JPT-M reached 22.57 mg L^−1^, representing remarkable increases of 159.42% and 1356.13% compared with the control strain JPT-25 and the original strain FH215-HBA, respectively. Compared with the *hemE* knockdown strain JPT-E1000 and the *cysG* knockdown strain JPT-G2000, HBA titer also increased to a certain extent. At the same time, the strain still maintained a good growth rate compared with the control (Fig. 6). We therefore achieved high HBA production while maintaining optimal cell growth by fine-tuning the biosynthetic pathway of the target product and its competing branching pathways.

## Discussion

With the development of synthetic biology and metabolic engineering, researchers began to introduce heterologous pathways into established production hosts, enabling them to produce a variety of highly valuable compounds [32–34]. However, incorrect metabolic engineering approaches often disrupt metabolic networks, trigger energetic inefficiencies inside the cell, and hinder growth [35]. Optimally flux through metabolic pathways is an important prerequisite for the production of various chemicals in industrial fermentations [36]. In order to balance the metabolism of a production host, it is necessary to optimize the metabolic pathway at different levels [36]. Through the precise regulation of gene expression by promoter engineering, it was possible to optimize the production of many different chemicals, such as lycopene and l-threonine [37, 38]. Designed ribosomal binding site libraries for pathway genes have been extensively applied to enhance the production of high-value-added compounds such as 5-aminolevulinic acid, carotenoids, and shikimic acid [39–41]. These examples confirm the importance of a balanced metabolic network for the production of valuable chemicals.

To increase the HBA yield of *E. coli* in this study, we introduced a heterologous urogen III biosynthesis pathway from *S. meliloti* 320. Different from the strategies adopted in previous studies on vitamin B_12_ biosynthesis, we used a combinatorial approach based on type-II restriction enzymes combined with designed ribosomal binding site libraries to efficiently screen out an optimal ratio of initial translation rates of the four *hemABCD* genes. Previously, Ye et al. constructed an optimized MVA pathway using similar methods, which doubled the production of β-carotene in *E. coli* [42]. Compared with the traditional strategy of optimizing each individual pathway gene one by one, this method can theoretically be used to screen all the possible combinations to achieve the maximum optimization effect [42]. In addition, we further optimized the expression of the pathway according to the law of combined expression strength. The results of RBS library screening indicated that a translation initiation ratio of 10:1:1:5 is optimal for the *hemABCD* genes. Notably, this ratio was similar to a previously reported optimal proportion of enzymes in vitro [43]. However, it is worth noting that the predicted TIR of an RBS cannot completely reflect the expression state of the pathway, but it can estimate the optimal overall expression trend. We noticed that the translation initiation rate of *hemA* in vivo is very high, which may be explained by the fact that the translation of *hemA* is inhibited by heme feedback [23]. Next, we used promoter optimization to coordinate the transcription levels of the two modules, which further increased the HBA titer. The yield of secondary metabolites can be further increased by fine-tuning the expression levels of genes in the pathway. More importantly, this fine regulation can reduce energy waste to some extent, which is of great significance for the production of secondary metabolites with long pathways.

Generally speaking, the introduction of foreign genes will inevitably lead to metabolic abnormalities, mainly the accumulation of intermediates and by-products [14]. In *E. coli*, the increase of urogen III levels can lead to an increase of heme and siroheme, whereby heme accumulation can become toxic and compromise cell viability [29]. Early work demonstrated that heme inhibits the enzymatic activity of ALAS in *Rhodobacter sphaeroides* [44]. Perhaps more importantly, these two pathways compete for precursors of the target products. However, these two competing pathways cannot be completely knocked out because they are essential. It has been reported that antisense RNA and small RNA strategies are effective approaches for knocking down the heme branch, but the effect was unstable and the inhibition level cannot be finely controlled [6, 12]. To overcome these shortcomings, we used the CRISPR/cas9 system to edit the *E. coli* genome and decreased the effects of the heme and siroheme branches by reducing the initial translation rates of *hemE* and *cysG*, respectively. As expected, the growth trend and HBA titer of the strain with *hemE* knockdown increased compared with the control strain. In contrast with the instability of the antisense RNA strategy [12], the heme levels of strains constructed via RBS engineered remained stable until the end of fermentation. Our results thus confirmed the feasibility of using RBS engineering to control the metabolic flux of the heme branch. However, we noticed that the HBA titer of strains with a blocked siroheme biosynthesis pathway decreased significantly, perhaps because we ignored the role of siroheme in cell growth. A more in-depth investigation of the function of *cysG* in future studies is therefore warranted. In this study, we obtained a recombinant *E. coli* strain with a high HBA titer through multi-level optimization of the precursor module, together with fine-tuning of competing biosynthetic pathways.

## Conclusions

We used a series of metabolic engineering strategies to optimize the HBA biosynthesis pathway, especially the metabolic network related to urogen III, and successfully increased the HBA titer of transgenic *E. coli*. Combinatorial optimization of RBS libraries for the genes involved in urogen III biosynthesis provided a sufficiently large precursor pool for HBA production. Through the precise regulation of the metabolic flux through the relevant branch, we eliminated the toxicity of heme accumulation, abrogating its negative effects on cell growth and product accumulation. In addition, fine-tuning of the competing siroheme branch also increased the HBA titer. Taken together, this study provides an example of effective pathway optimization using multi-level metabolic engineering strategies, and lays a foundation for the industrial production of vitamin B_12_ in *E. coli*.

## Supplementary information

**Additional file 1: Table S1.** Strains and plasmids used in this study. **Table S2.** Primers used in this study. **Table S3.** RBS sequences involved in this study. **Table S4.** RBS sequences with their calculated strength of representative strains from the combinatory expression library. **Fig. S1**. Fluorescence intensity of recombinant strains undergoing artificial regulation of gene expression in the precursor module. **Fig. S2.** The fluorescence intensity of recombinant strains expressing precursor modules driven by different promoters. **Fig. S3.** qRT-PCR determination of *hemABCD* expression driven by different promoters.

## Data Availability

All data generated and analyzed during this study are included in this manuscript and in its Additional file 1.

## Abbreviations

HBA: Hydrogenobyrinic acid
Urogen III: Uroporphyrinogen III
ALA: 5-Aminolevulinic acid
SAM: *S*-adenosyl-l-methionine
PBG: Porphobilinogen
RBS: Ribosome binding site
HPLC: High performance liquid chromatography
ALAS: 5-Aminolevulinic acid synthase
PBGS: Porphobilinogen synthase
PBGD: Porphobilinogen deaminase
UROS: Uroporphyrinogen III synthase
SUMT: S-adenosyl-l-methionine dependent uroporphyrinogen III methyltransferase
TIRs: Translational initiation rates
UROD: Uroporphyrinogen III decarboxylase
