# Supplementary material for: Optimization of hydrogenobyrinic acid biosynthesis in Escherichia coli using multi-level metabolic engineering strategies
Source: Microb Cell Fact. 2020 Jun 1;19:118. doi: 10.1186/s12934-020-01377-2 (PMC7268678; doi:10.1186/s12934-020-01377-2)
Supplement: Supplementary file 1 — Additional file 1: Table S1. Strains and plasmids used in this study. Table S2. Primers used in this study. Table S3. RBS sequences involved in this study. Table S4. RBS sequences with their calculated strength of representative strains from the combinatory expression library. Fig. S1. Fluorescence intensity of recombinant strains undergoing artificial regulation of gene expression in the precursor module. Fig. S2. The fluorescence intensity of recombinant strains expressing precursor modules driven by different promoters. Fig. S3. qRT-PCR determination of hemABCD expression driven by different promoters. [file 12934_2020_1377_MOESM1_ESM.doc]

**Additional file 1**

**Optimization of hydrogenobyrinic acid biosynthesis in *Escherichia coli* using multi-level metabolic engineering strategies**

Pingtao Jiang^1,2†^, Huan Fang^2,3†^, Jing Zhao^2,4^, Huina Dong^2^, Zhaoxia Jin^1*^, Dawei Zhang^2,3,4*^

*Correspondence: jinzx2018@163.com; zhang_dw@tib.cas.cn.

†Pingtao Jiang and Huan Fang contributed equally to this work

^1^ School of Biological Engineering, Dalian Polytechnic University, Dalian 116034, China.

^2^ Tianjin Institute of Industrial Biotechnology, Chinese Academy of Sciences, Tianjin 300308, China.

^3^ Key Laboratory of Systems Microbial Biotechnology, Chinese Academy of Sciences, Tianjin 300308, China.

^4^ University of Chinese Academy of Sciences, Beijing 100049, China.

**
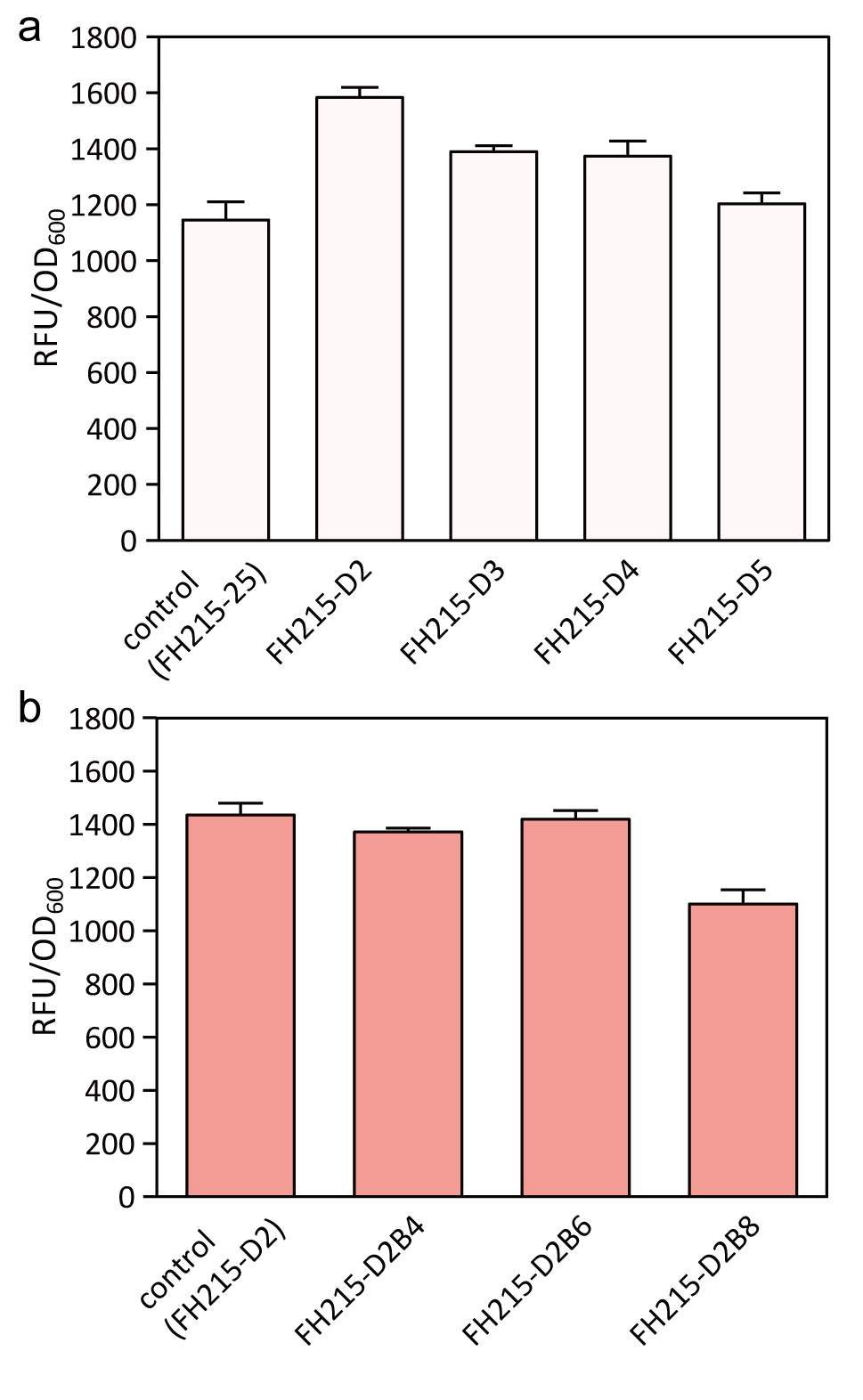
**

**Fig. S1 Fluorescence intensity of recombinant strains undergoing artificial regulation of gene expression in the precursor module.** The production of urogen III of each strain was indirectly quantified by measuring the fluorescence intensity of sirohydrochlorin. **a** and **b** show the fluorescence status of the recombinant strains after regulating the initial translation efficiency of *hemD* and *hemB*, respectively. Error bars indicate standard deviations from three biological replicates.


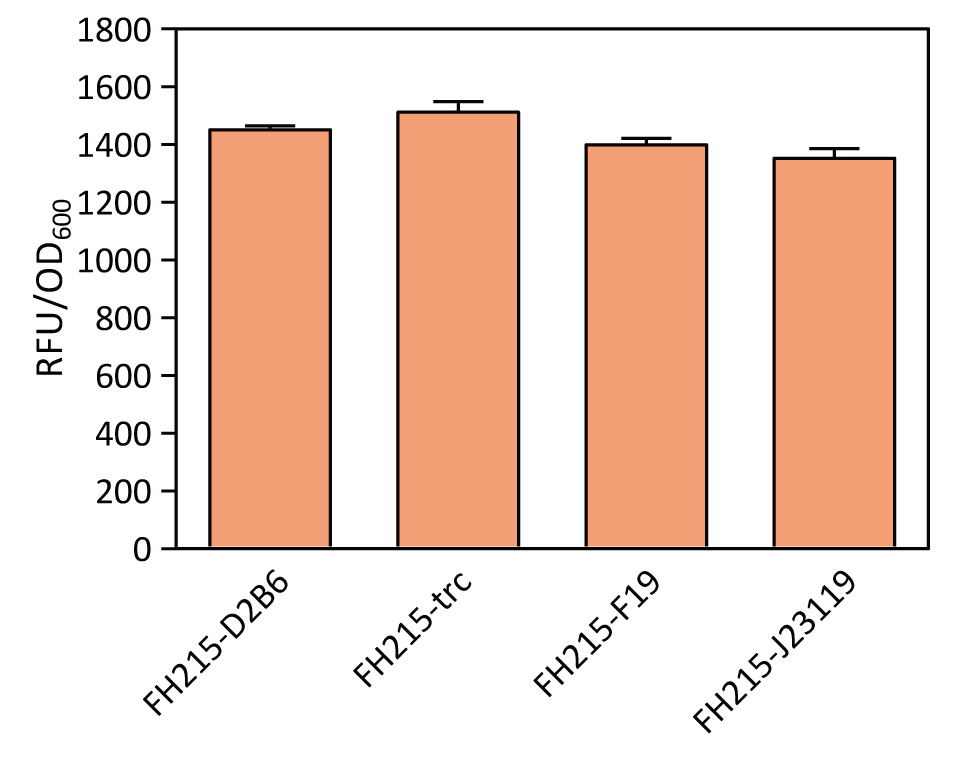


**Fig. S2** **The fluorescence intensity of recombinant strains expressing precursor modules driven by different promoters.** The histogram shows the fluorescence state of the recombinant strains with different promoters driving the precursor modules. The tac promoter was used in the control strain FH215-D2B6, while the trc, F19, and J23119 promoters were used in the recombinant strains FH215-trc, FH215-F19 and FH215-J23119, respectively. Error bars indicate standard deviations from three biological replicates.


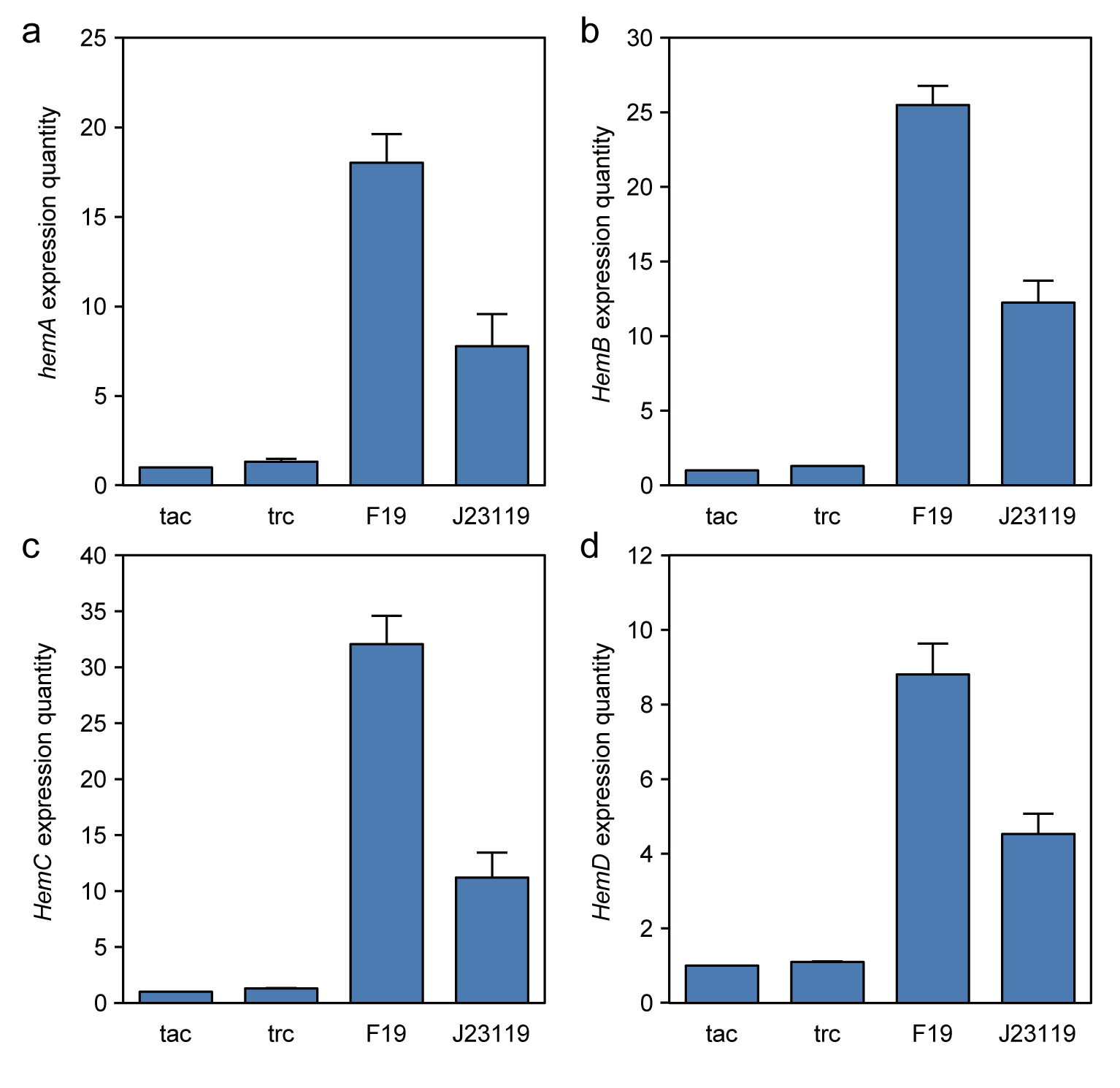


**Fig. S3** **qRT-PCR determination of *hemABCD* expression levels driven by different promoters.** The RNA expression levels of *hemA*, *hemB*, *hemC* and *hemD* in the engineered strains FH215-D2B6, FH215-trc, FH215-F19 and FH215-J23119 were measured by qRT-PCR. The relative transcriptional intensity of each gene in the recombinant strains FH215-D2B6, FH215-trc, FH215-F19 and FH215-J23119 was normalized to the control strain FH215-D2B6. Error bars indicate standard deviations from three biological replicates.

**Table S1**. Strains and plasmids used in this study

| **Plasmid and strain** | **Description** | **Reference or source** |
| --- | --- | --- |
| **Plasmid** |  |  |
| pET28a | Expression vector; T7 promoter; pBR322 ori; Kan^r^ | Novagen |
| p15ASI | Expression vector; tac and lacUV5 promoter; p15a ori; Cm^r^ | [1] |
| pRed_Cas9 | pKD46 derivative containing Cas9 for genome editing | [2] |
| pET28a-hemB | pET28a derivative containing hemB fromSinorhizobiummeliloti 320; Kan^r^ | [3] |
| pET28a-hemC | pET28a derivative containing hemC fromSinorhizobiummeliloti 320; Kan^r^ | [3] |
| pET28a-hemD | pET28a derivative containing hemD fromSinorhizobiummeliloti 320; Kan^r^ | [3] |
| p15ASI-SmhemA | p15ASI derivative containing hemA fromSinorhizobiummeliloti 320; Cm^r^ | This study |
| p15ASI-SmhemA-hemABCD | p15ASI-SmhemA derivative containing hemA, hemB, hemC and hemD from S. meliloti 320.Each gene is preceded by a RBS library; Cm^r^ | This study |
| pET28-HBA | pET28a derivative containing cobAIGJMFKLH; Kan^r^ | [1] |
| p15ASI-10 | A variant ofp15ASI-SmhemA-hemABCD; The characteristics of RBS sequence are shown in Table 3; Cm^r^ | This study |
| p15ASI-18 | A variant of p15ASI-SmhemA-hemABCD; The characteristics of RBS sequence are shown in Table 3; Cm^r^ | This study |
| p15ASI-25 | A variant ofp15ASI-SmhemA-hemABCD; The characteristics of RBS sequence are shown in Table 3; Cm^r^ | This study |
| p15ASI-29 | A variant of p15ASI-SmhemA-hemABCD; The characteristics of RBS sequence are shown in Table 3; Cm^r^ | This study |
| p15ASI-66 | A variant of p15ASI-SmhemA-hemABCD; The characteristics of RBS sequence are shown in Table 3; Cm^r^ | This study |
| p15ASI-93 | A variant of p15ASI-SmhemA-hemABCD; The characteristics of RBS sequence are shown in Table 3; Cm^r^ | This study |
| p15ASI-113 | A variant ofp15ASI-SmhemA-hemABCD; The characteristics of RBS sequence are shown in Table 3; Cm^r^ | This study |
| p15ASI-121 | A variant of p15ASI-SmhemA-hemABCD; The characteristics of RBS sequence are shown in Table 3; Cm^r^ | This study |
| p15ASI-122 | A variant ofp15ASI-SmhemA-hemABCD; The characteristics of RBS sequence are shown in Table 3; Cm^r^ | This study |
| p15ASI-181 | A variant of p15ASI-SmhemA-hemABCD; The characteristics of RBS sequence are shown in Table 3; Cm^r^ | This study |
| p15ASI-25D2 | Replace the RBS sequence of hemD with D2 in p15ASI-25; Cm^r^ | This study |
| p15ASI-25D3 | Replace the RBS sequence of hemD with D3 in p15ASI-25; Cm^r^ | This study |
| p15ASI-25D4 | Replace the RBS sequence of hemD with D4 in p15ASI-25; Cm^r^ | This study |
| p15ASI-25D5 | Replace the RBS sequence of hemD with D5 in p15ASI-25; Cm^r^ | This study |
| p15ASI-25D2B4 | Replace the RBS sequence of hemB with B4 in p15ASI-25D2; Cm^r^ | This study |
| p15ASI-25D2B6 | Replace the RBS sequence of hemB with B6 in p15ASI-25D2; Cm^r^ | This study |
| p15ASI-25D2B8 | Replace the RBS sequence of hemB with B8 in p15ASI-25D2; Cm^r^ | This study |
| p15ASI-25trc | Replace the tac promoter in p15ASI-25D2B6 with trc promoter; Cm^r^ | This study |
| p15ASI-25pF19 | Replace the tac promoter in p15ASI-25D2B6 with pF19 promoter; Cm^r^ | This study |
| p15ASI-25pJ23119 | Replace the tac promoter in p15ASI-25D2B6 with pJ23119 promoter; Cm^r^ | This study |
| **Strain** |  |  |
| *E. coli* MG1655 | F- λ- ilvG- rfb-50 rph-1 | ATCC |
| *E. coli* DH5α | Escherichia coli strain used in cloning | Invitrogen |
| *Sinorhizobiummeliloti* 320 | Source of vitamin B_12_ biosynthetic pathway genes | [4] |
| MG1655 (DE3) | E. coli MG1655 λ(DE3) | [1] |
| FH215 | MG1655(DE3)△ldhA:: PTac-cbiMNQO△endA | [1] |
| FH215-HBA | FH215 with pET28-HBA | This study |
| FH215DEcysG | Knockout of gene cysG in FH215 | This study |
| FH215E100 | Replace the RBS sequence of hemE with E100 in FH215 | This study |
| FH215E500 | Replace the RBS sequence of hemE with E500 in FH215 | This study |
| FH215E1000 | Replace the RBS sequence of hemE with E1000 in FH215 | This study |
| FH215E2000 | Replace the RBS sequence of hemE with E2000 in FH215 | This study |
| FH215G100 | Replace the RBS sequence of cysG with G100 in FH215 | This study |
| FH215G500 | Replace the RBS sequence of cysG with G500 in FH215 | This study |
| FH215G2000 | Replace the RBS sequence of cysG with G2000 in FH215 | This study |
| FH215-E1G2 | Replace the RBS sequence of cysG with G2000 in FH215E1000 | This study |
| JPT10 | FH215 with pET28-HBA and p15ASI-10 | This study |
| JPT18 | FH215 with pET28-HBA and p15ASI-18 | This study |
| JPT25 | FH215 with pET28-HBA and p15ASI-25 | This study |
| JPT29 | FH215 with pET28-HBA and p15ASI-29 | This study |
| JPT66 | FH215 with pET28-HBA and p15ASI-66 | This study |
| JPT93 | FH215 with pET28-HBA and p15ASI-93 | This study |
| JPT113 | FH215 with pET28-HBA and p15ASI-113 | This study |
| JPT121 | FH215 with pET28-HBA and p15ASI-121 | This study |
| JPT122 | FH215 with pET28-HBA and p15ASI-122 | This study |
| JPT183 | FH215 with pET28-HBA and p15ASI-183 | This study |
| JPT25D2 | FH215 with pET28-HBA and p15ASI-25D2 | This study |
| JPT25D3 | FH215 with pET28-HBA and p15ASI-25D3 | This study |
| JPT25D4 | FH215 with pET28-HBA and p15ASI-25D4 | This study |
| JPT25D5 | FH215 with pET28-HBA and p15ASI-25D5 | This study |
| JPT25D2B4 | FH215 with pET28-HBA and p15ASI-25D2B4 | This study |
| JPT25D2B6 | FH215 with pET28-HBA and p15ASI-25D2B6 | This study |
| JPT25D2B8 | FH215 with pET28-HBA and p15ASI-25D2B8 | This study |
| JPT25-trc | FH215 with pET28-HBA and p15ASI-25trc | This study |
| JPT25-pF19 | FH215 with pET28-HBA and p15ASI-25pF19 | This study |
| JPT25-pJ23119 | FH215 with pET28-HBA and p15ASI-pJ23119 | This study |
| FH215-D2 | FH215 with p15ASI-25D2 | This study |
| FH215-D3 | FH215 with p15ASI-25D3 | This study |
| FH215-D4 | FH215 with p15ASI-25D4 | This study |
| FH215-D5 | FH215 with p15ASI-25D5 | This study |
| FH215-D2B4 | FH215 with p15ASI-25D2B4 | This study |
| FH215-D2B6 | FH215 with p15ASI-25D2B6 | This study |
| FH215-D2B8 | FH215 with p15ASI-25D2B8 | This study |
| FH215-trc | FH215 with p15ASI-25trc | This study |
| FH215-F19 | FH215 with p15ASI-25pF19 | This study |
| FH215-J23119 | FH215 with p15ASI-pJ23119 | This study |
| JPT-E100 | FH215E100 with pET28-HBA and p15ASI-25D2B6 | This study |
| JPT-E500 | FH215E500 with pET28-HBA and p15ASI-25D2B6 | This study |
| JPT-E1000 | FH215E1000 with pET28-HBA and p15ASI-25D2B6 | This study |
| JPT-E2000 | FH215E2000 with pET28-HBA and p15ASI-25D2B6 | This study |
| JPT-DEcysG | FH215DEcysG with pET28-HBA and p15ASI-25D2B6 | This study |
| JPT-G100 | FH215G100 with pET28-HBA and p15ASI-25D2B6 | This study |
| JPT-G500 | FH215G500 with pET28-HBA and p15ASI-25D2B6 | This study |
| JPT-G2000 | FH215G2000 with pET28-HBA and p15ASI-25D2B6 | This study |
| JPT-M | FH215E1G2 with pET28-HBA and p15ASI-25pF19 | This study |

1. Fang H, Li D, Kang J, Jiang P, Sun J, Zhang D: **Metabolic engineering of Escherichia coli for de novo biosynthesis of vitamin B12.***Nature Communications* 2018, **9**.

2. Zhao D, Yuan S, Xiong B, Sun H, Ye L, Li J, Zhang X, Bi C: **Development of a fast and easy method for Escherichia coli genome editing with CRISPR/Cas9.***Microbial Cell Factories* 2016, **15**.

3. Fang H, Dong H, Cai T, Zheng P, Li H, Zhang D, Sun J: **In Vitro Optimization of Enzymes Involved in Precorrin-2 Synthesis Using Response Surface Methodology.***PLOS ONE* 2016, **11:**e0151149.

4. Dong H, Li S, Fang H, Xia M, Zheng P, Zhang D, Sun J: **A newly isolated and identified vitamin B12 producing strain: Sinorhizobium meliloti 320.***Bioprocess and Biosystems Engineering* 2016, **39**.

**Table S2**. Primers used in this study

| Designation | Sequence (5` to 3`) | Description |
| --- | --- | --- |
| Pet28-hemC-mut-F | GGTGGCGAGGCCGGCGATCGGCGTGCGGCACGAGCCG | Removing the *Bsa*I restriction site in *hemC* |
| Pet28-hemC-mut-R | ATCGCCGGCCTCGCCACCTCGGACGGCACCCATCTC | Removing the *Bsa*I restriction site in *hemC* |
| Pet28-hemB-mut-F | AGCGGCCGGCCTCGGCATCCCGGCGATCGCCACCTTCC | Removing the *Bsa*I restriction site in *hemB* |
| Pet28-hemB-mut-R | GATGCCGAGGCCGGCCGCTTCCTTTACCGCTTCCACGGCC | Removing the *Bsa*I restriction site in *hemB* |
| Pet28-hemD-mut-F1 | CTCGCGAGAAACCGCGCGACAGTTCGTTCGCCTGCTTTCCG | Removing the*Bsa*I restriction site in *hemD* |
| Pet28-hemD-mut-R1 | TCGCGCGGTTTCTCGCGAGTATAAAAGCACGGCGTCGGGACG | Removing the*Bsa*I restriction site in *hemD* |
| Pet28-hemD-mut-F2 | CGTGGCGGAAACCGCCGCAAGCACCGATGAAGACAGCCTTTTC | Removing the*Bsa*I restriction site in *hemD* |
| Pet28-hemD-mut-R2 | TGCGGCGGTTTCCGCCACGACGTTACCCGGCAGTGCCTC | Removing the*Bsa*I restriction site in *hemD* |
| SmcobA-P15asi-F-XhoI | CCGCTCGAGTCAAATAAGGAGGTATTATTATGATCGACGACCTCTTTGC | *SmcobA* gene cloning |
| SmcobA-P15asi-R-KpnI | GGGGTACCTCATGCCGGGTTCCTGAG | *SmcobA* gene cloning |
| P15ASISmcobA-F-golden | CCAGGTCTCAGATGCCCAGGCATCAAATAAAACG | Construction of *hemA, hemB, hemC and hemD* expression plasmid |
| P15ASISmcobA-R-golden | CCAGGTCTCATGGGATCCGAATTCCTGCAG | Construction of *hemA, hemB, hemC and hemD* expression plasmid |
| hemC-F-golden | CCAGGTCTCAGACCCTCAGCKAAATAWRGAGGATGGAATGCAAACAAAACCTTTCCGG | *hemC* gene cloning |
| hemC-R-golden | CCAGGTCTCACGTTAAGTCCAGCTTGCAAAGAAG | *hemC* gene cloning |
| hemD-F-golden | CCAGGTCTCAAACGTACTGTCCKAASGWGGTARTAAAATGCGCGTGCTCGTCAC | *hemD* gene cloning |
| hemD-R-golden | CCAGGTCTCACTCGTTAGAGAAGACTGAAAAGGCTGTCTTC | *hemD* gene cloning |
| hemA-F-golden | CCAGGTCTCACCTATAACATACABTTAAGRAGRACTAATGATTGAAGAAAGCGGCCGG | *hemA* gene cloning |
| hemA-R-golden | CCAGGTCTCAGAATTACGCGACAGCGCGGGC | *hemA* gene cloning |
| hemB-F-golden | CCAGGTCTCAGTAARCGCACATTTTAARGAGGKCTCAATaTGGACAGGGTCACCGGC | *hemB* gene cloning |
| hemB-R-golden | CCAGGTCTCACATCAGCGGCCTTTGGCGAG | *hemB* gene cloning |
| pF19-hemABCD-F | AGGATATGCCTTGATATAATGCTAGCTGTGGAATTGTGAGCGGATAAC | Modifying the promoter of JPT25D2B6 |
| pF19-hemABCD-R | TATATCAAGGCATATCCTACATGTCAACCTAATGCAGGAGTCGCAT | Modifying the promoter of JPT25D2B6 |
| pJ23119-hemABCD-F | AGCTCAGTCCTAGGTATAATGCTAGCTGTGGAATTGTGAGCGGATAAC | Modifying the promoter of JPT25D2B6 |
| pJ23119-hemABCD-R | TATACCTAGGACTGAGCTAGCTGTCAACCTAATGCAGGAGTCGCAT | Modifying the promoter of JPT25D2B6 |
| P15ASI-Trc-F-New | ATCATCCGGCTCGTATAATGTGTGGAATTGTGAGCGGATAAC | Modifying the promoter of JPT25D2B6 |
| P15ASI-Trc-R-New | ATTATACGAGCCGGATGATTAATTGTCAACCTAATGCAGGAG | Modifying the promoter of JPT25D2B6 |
| hemE-F-PstI | AACTGCAGGTAAGCCACGAGCAGATG | *hemE* gene cloning |
| hemE-R-BamHI | CGGGATCCGAGCGAGAACTGTTGATAATC | *hemE* gene cloning |
| Pcas9-1-R-hemE-golden | CCAGGTCTCAAAACAGGCCCATGGATTCT | Construction of hemE weakening plasmid |
| Pcas9-2-F-hemE-golden | CCAGGTCTCAGCTCTGAATGGAAGCTTGGATTCTC | Construction of hemE weakening plasmid |
| Pcas9-1-F-hemE-golden | CCAGGTCTCACCTGACGCACTAGTTTTAGAGCTAGAAATAGCAAG | Construction of hemE weakening plasmid |
| Pcas9-2-R-hemE-golden | CCAGGTCTCACAGGCGGTCAGTGCTAAGATCTGACTCCATAAC | Construction of hemE weakening plasmid |
| hemE-F-golden | CCAGGTCTCAGTAAGCCACGAGCAGATG | Construction of hemE weakening plasmid |
| hemE-R-golden | CCAGGTCTCAGAGCGAGAACTGTTGATAATC | Construction of hemE weakening plasmid |
| YZ-hemE-F | CATCGTAACGGTGTCCATA | colony PCR |
| YZ-hemE-R | ACTCCTTAGCGGTGATACT | colony PCR |
| hemE-F-100 | ATACTGGGATAATGACCGAACTTAAAAACGATCG | Modifying RBS of h*emE* |
| hemE-R-100 | TACGTGTCCGCTCATCACTCATACTCTGCC | Modifying RBS of *hemE* |
| hemE-F-500 | ATAACACTGGTTATGACCGAACTTAAAAACGATCG | Modifying RBS of *hemE* |
| hemE-R-500 | TGAAAAAGTTTCCTCACTCATACTCTGCCCG | Modifying RBS of *hemE* |
| hemE-F-1000 | TGGGAAATCGGAAATGACCGAACTTAAAAACGATCG | Modifying RBS of *hemE* |
| hemE-R-1000 | GCCTGTGTTGGCTCACTCATACTCTGCCCG | Modifying RBS of *hemE* |
| hemE-F-2000 | TGAGGGAGCGGCATGACCGAACTTAAAAACGATCG | Modifying RBS of *hemE* |
| hemE-R-2000 | GTGTGTTTCTAGGTCACTCATACTCTGCCCG | Modifying RBS of *hemE* |
| cysG-UP-F-golden | CCAGGTCTCAGTCGGTAGATACCAGCATCGTTC | Construction of cysG weakening plasmid |
| cysG-UP-R-golden | TTAACCGGCAGCCGTTTC | Construction of cysG weakening plasmid |
| cysG-DN-F-golden | GTGGATCATTTGCCTATATTTTGCC | Construction of cysG weakening plasmid |
| cysG-DN-R-golden | CCAGGTCTCACATACCATGAGCGGTGAGCG | Construction of cysG weakening plasmid |
| cysG-UP-R-100-overlap | AAGGGTCAATGGTGAGCCGGCGTAGTTAACCGGCAGCCGTTTC | Modifying RBS of *cysG* |
| cysG-DN-F-100-overlap | CTACGCCGGCTCACCATTGACCCTTGTGGATCATTTGCCTATATTTTGCC | Modifying RBS of *cysG* |
| cysG-UP-R-500-overlap | TCGGCAACTCCGAGGGGCAGCGGTTCTTAACCGGCAGCCGTTTC | Modifying RBS of *cysG* |
| cysG-DN-F-500-overlap | GAACCGCTGCCCCTCGGAGTTGCCGAGTGGATCATTTGCCTATATTTTGCC | Modifying RBS of *cysG* |
| cysG-UP-R-2000-overlap | GGTTTTTTTTACCCCTATGCCTGCTTAACCGGCAGCCGTTTC | Modifying RBS of *cysG* |
| cysG-DN-F-2000-overlap | GCAGGCATAGGGGTAAAAAAAACCGTGGATCATTTGCCTATATTTTGCC | Modifying RBS of *cysG* |
| Pcas9-1-R-cysG-golden | CCAGGTCTCACGACAGGCCCATGGATTCT | Construction of cysG weakening plasmid |
| Pcas9-2-F-cysG-golden | CCAGGTCTCATATGAATGGAAGCTTGGATTCTC | Construction of cysG weakening plasmid |
| Pcas9-1-F-cysG-golden | CCAGGTCTCAGTTAATTACTAAGTTTTAGAGCTAGAAATAGCAAG | Construction of cysG weakening plasmid |
| Pcas9-2-R-cysG-golden | CCAGGTCTCATAACCGGCAGCCGCTAAGATCTGACTCCATAAC | Construction of cysG weakening plasmid |
| YZ-cysG-genome-F | GCTATCTGGTGGTGTCTG | colony PCR |
| YZ-cysG-genome-R | TGGCATTCCGTGTTCAAT | colony PCR |
| QPCR-16S-F | ATCAGAATGCCACGGTGAATAC | qRT-PCR for 16S sRNA |
| QPCR-16S-R | CTACGGTTACCTTGTTACGACTT | qRT-PCR for 16S sRNA |
| QPCR-hemA-F | AACCATTACCACGTCCTGCTTGAG | qRT-PCR for *hemA* |
| QPCR-hemA-R | TCAGCGACCGAATTATGCTTGAAGA | qRT-PCR for *hemA* |
| QPCR-hemB-F | ACCAGAATGTCGGCATCATGTCCTA | qRT-PCR for *hemB* |
| QPCR-hemB-R | TCCGCCAGCAGATGTCGAGATAG | qRT-PCR for *hemB* |
| QPCR-hemC-F | CTTCATCGGTCGGACGGCAAA | qRT-PCR for *hemC* |
| QPCR-hemC-R | ATAGGCAAGCAGTGTCGCATCG | qRT-PCR for *hemC* |
| QPCR-hemD-F | GACACATCTTGCTGCCGTCTCTG | qRT-PCR for *hemD* |
| QPCR-hemD-R | CGATGGTCGCACCGATCAGTTC | qRT-PCR for *hemD* |

**Table S3**. RBS sequences involved in this study

| Designation | Sequence (5` to 3`) | RBS TIR |
| --- | --- | --- |
| *hemE* |  |  |
| E100 | TGAGCGGACACGTAATACTGGGATA | 96.69034944 |
| E500 | GGAAACTTTTTCAATAACACTGGTT | 508.9548729 |
| E1000 | GCCAACACAGGCTGGGAAATCGGAA | 1016.777791 |
| E2000 | CCTAGAAACACACTGAGGGAGCGGC | 1999.547854 |
| *cysG* |  |  |
| G100 | CTACGCCGGCTCACCATTGACCCTT | 104.31 |
| G500 | GAACCGCTGCCCCTCGGAGTTGCCGA | 558.87 |
| G2000 | GCAGGCATAGGGGTAAAAAAAACC | 1997.34 |
| *hemA* |  |  |
| A1 | TATAACATACACTTAAGGAGGACTA | 104944.2544 |
| A2 | TATAACATACATTTAAGGAGGACTA | 60878.19356 |
| A3 | TATAACATACAGTTAAGGAGGACTA | 34841.84559 |
| A4 | TATAACATACACTTAAGAAGGACTA | 9881.785756 |
| A5 | TATAACATACATTTAAGAAGGACTA | 6050.520679 |
| A6 | TATAACATACAGTTAAGAAGGACTA | 3589.783807 |
| A7 | TATAACATACATTTAAGAAGAACTA | 1786.971615 |
| A8 | TATAACATACAGTTAAGAAGAACTA | 943.1390153 |
| *hemB* |  |  |
| B1 | ACGCACATTTTAAGGAGGTCTCAAT | 67233.92823 |
| B2 | GCGCACATTTTAAGGAGGTCTCAAT | 50249.3759 |
| B3 | GCGCACATTTTAAGGAGGGCTCAAT | 28629.62072 |
| B4 | ACGCACATTTTAAGGAGGGCTCAAT | 16511.17151 |
| B5 | ACGCACATTTTAAAGAGGTCTCAAT | 6134.560904 |
| B6 | GCGCACATTTTAAAGAGGTCTCAAT | 4584.855667 |
| B7 | GCGCACATTTTAAAGAGGGCTCAAT | 2612.225617 |
| B8 | ACGCACATTTTAAAGAGGGCTCAAT | 1568.785958 |
| *hemC* |  |  |
| C1 | CCCTCAGCGAAATAAGGAGGATGGA | 71928.49833 |
| C2 | CCCTCAGCTAAATAAGGAGGATGGA | 28849.16626 |
| C3 | CCCTCAGCGAAATATGGAGGATGGA | 14556.1433 |
| C4 | CCCTCAGCGAAATAAAGAGGATGGA | 6445.817541 |
| C5 | CCCTCAGCTAAATATGGAGGATGGA | 5997.990879 |
| C6 | CCCTCAGCGAAATATAGAGGATGGA | 4357.473316 |
| C7 | CCCTCAGCTAAATAAAGAGGATGGA | 2585.29604 |
| C8 | CCCTCAGCTAAATATAGAGGATGGA | 1747.700495 |
| *hemD* |  |  |
| D1 | AAAAAATATACGAGGAGGTTTTCAT | 87827.02205 |
| D2 | AAAAAATCTACGAGGAGGTTTTCAT | 34133.35196 |
| D3 | AAAAAATATACGAGGAGTTTTTCAT | 19273.23591 |
| D4 | AAAAAATCTACGAGGAGTTTTTCAT | 7064.747241 |
| D5 | AAAAAATATACGAGTAGGTTTTCAT | 3243.132188 |
| D6 | AAAAAATCTACGAGTAGGTTTTCAT | 1294.918547 |
| D7 | AAAAAATATACGAGTAGTTTTTCAT | 763.5548423 |
| D8 | AAAAAATCTACGAGTAGTTTTTCAT | 484.6560164 |

| **Table S4.** RBS sequences with their calculated strength of representative strains from the combinatory expression library | | | | | | | | | | | |
| --- | --- | --- | --- | --- | --- | --- | --- | --- | --- | --- | --- |
|  | The genes RBS of urogen III biosynthesis pathway | | | | | | | | | | |
| trains | *hemA* | |  | *hemB* | |  | *hemC* | |  | *hemD* | |
|  | Sequence | T.I.R* |  | Sequence | T.I.R |  | Sequence | T.I.R |  | Sequence | T.I.R |
| JPT10 | TATAACATACACTTAAGAAGGACTA | 9881.79 |  | GCGCACATTTTAAAGAGGGCTCAAT | 2612.23 |  | CCCTCAGCTAAATATAGAGGATGGA | 1747.70 |  | AAAAAATCTACGAGGAGGTTTTCAT | 34133.35 |
| JPT18 | TATAACATACATTTAAGAAGGACTA | 6050.52 |  | GCGCACATTTTAAAGAGGTCTCAAT | 4584.86 |  | CCCTCAGCTAAATATAGAGGATGGA | 1747.70 |  | AAAAAATCTACGAGGAGGTTTTCAT | 34133.35 |
| JPT25 | TATAACATACATTTAAGGAGGACTA | 60878.19 |  | GCGCACATTTTAAGGAGGGCTCAAT | 28629.62 |  | CCCTCAGCTAAATATGGAGGATGGA | 5997.99 |  | AAAAAATCTACGAGTAGGTTTTCAT | 1294.92 |
| JPT66 | TATAACATACACTTAAGAAGGACTA | 9881.79 |  | GCGCACATTTTAAGGAGGGCTCAAT | 28629.62 |  | CCCTCAGCTAAATAAAGAGGATGGA | 2585.30 |  | AAAAAATATACGAGTAGGTTTTCAT | 3243.13 |
| JPT121 | TATAACATACACTTAAGAAGGACTA | 9881.79 |  | GCGCACATTTTAAAGAGGTCTCAAT | 4584.86 |  | CCCTCAGCTAAATATGGAGGATGGA | 5997.99 |  | AAAAAATCTACGAGGAGTTTTTCAT | 7064.75 |
| JPT29 | TATAACATACAGTTAAGAAGGACTA | 3011.91 |  | ACGCACATTTTAAGGAGGTCTCAAT | 67233.93 |  | CCCTCAGCTAAATAAAGAGGATGGA | 2585.30 |  | AAAAAATCTACGAGTAGTTTTTCAT | 484.66 |
| JPT93 | TATAACATACAGTTAAGAAGAACTA | 943.14 |  | ACGCACATTTTAAGGAGGGCTCAAT | 16511.17 |  | CCCTCAGCTAAATATAGAGGATGGA | 1747.70 |  | AAAAAATATACGAGTAGGTTTTCAT | 3243.13 |
| JPT113 | TATAACATACAGTTAAGGAGGACTA | 34841.85 |  | GCGCACATTTTAAAGAGGGCTCAAT | 2612.23 |  | CCCTCAGCTAAATATAGAGGATGGA | 1747.70 |  | AAAAAATCTACGAGTAGGTTTTCAT | 1294.92 |
| JPT122 | TATAACATACAGTTAAGAAGAACTA | 943.14 |  | GCGCACATTTTAAAGAGGTCTCAAT | 4584.86 |  | CCCTCAGCGAAATATGGAGGATGGA | 14556.14 |  | AAAAAATATACGAGTAGTTTTTCAT | 763.55 |
| JPT183 | TATAACATACATTTAAGAAGGACTA | 6050.52 |  | ACGCACATTTTAAAGAGGTCTCAAT | 6134.56 |  | CCCTCAGCGAAATATAGAGGATGGA | 4357.47 |  | AAAAAATATACGAGTAGTTTTTCAT | 763.55 |
| *T.I.R: translation initiation rate, was calculated by RBS Library Calculator. | | | | | | | | | | | |
